# Hot-Melt Extrusion Drug Delivery System-Formulated *Haematococcus pluvialis* Extracts Regulate Inflammation and Oxidative Stress in Lipopolysaccharide-Stimulated Macrophages

**DOI:** 10.3390/md22110512

**Published:** 2024-11-13

**Authors:** Tae-Young Gil, Ha-Yeon Sim, Ha-Yeon Lee, Suji Ryu, Jong-Suep Baek, Dae Geun Kim, Jaehoon Sim, Hyo-Jin An

**Affiliations:** 1 Department of Oriental Pharmaceutical Science, College of Pharmacy, Kyung Hee University, Seoul 02447, Republic of Korea; sophia14t@gmail.com; 2 Department of Integrated Drug Development and Natural Products, Graduate School, Kyung Hee University, Seoul 02447, Republic of Korea; hysim6424@naver.com; 3 Department of Bio-Health Convergence, Kangwon National University, Chuncheon 24341, Republic of Korea; lhy101625@kangwon.ac.kr (H.-Y.L.); 202016297@kangwon.ac.kr (S.R.); jsbaek@kangwon.ac.kr (J.-S.B.); 4 BeNatureBioLab, Chuncheon 24206, Republic of Korea; 5 LED Agri-bio Fusion Technology Research Center (LAFTRC), Jeonbuk National University, Jeonbuk 54596, Republic of Korea; aings@jbnu.ac.kr; 6 Department of Pharmacy, College of Pharmacy, Kyung Hee University, Seoul 02447, Republic of Korea; jsim@khu.ac.kr

**Keywords:** hot-melt extrusion-drug delivery system, *Haematococcus pluvialis*, astaxanthin, inflammation, oxidative stress

## Abstract

*Haematococcus pluvialis* contains valuable bioactive compounds, including astaxanthin, proteins, and fatty acids. Astaxanthin is known for its various health benefits, such as preserving the redox balance and reducing inflammation. However, its low stability and poor water solubility present challenges for various applications. Hot-melt extrusion (HME) technology enhances the aqueous solubility of *H. pluvialis* extracts, increasing the usable astaxanthin content through nanoencapsulation (HME-DDS-applied extracts, ASX-60F and ASX-100F). This study compared the effects of HME-DDS-derived extracts (ASX-60F and ASX-100F) and the non-applied extract (ASX-C) under inflammatory and oxidative stress conditions. In animal models of sepsis, 60F and 100F treatment exhibited higher survival rates and a lower expression of pro-inflammatory biomarkers compared to those treated with C. In lipopolysaccharide-stimulated RAW 264.7 macrophages, nitric oxide (NO) production and the expression of pro-inflammatory mediators such as cyclooxygenase-2 and inducible NO synthase were reduced by 60F or 100F treatments via ERK/p-38 mitogen-activated protein kinase (MAPK) signaling. Moreover, 60F or 100F inhibited reactive oxygen species production regulated by nuclear factor erythroid 2-related factor 2 (Nrf2)/heme oxygenase-1 (HO-1) signaling. Collectively, these findings suggest that HME-DDS-derived *H. pluvialis* extracts exert anti-inflammatory and antioxidant effects by inhibiting MAPK phosphorylation and activating Nrf2/HO-1 expression.

## 1. Introduction

Sepsis is a severe, life-threatening inflammatory condition and a leading cause of mortality and morbidity [1]. It leads to systemic organ failure and immune dysfunction, including symptoms of systemic inflammatory response syndrome [2]. While inflammation is as an essential part of the innate defense system of the body against environmental injuries or pathogens, excessive inflammation contributes to tissue damage, provoking different diseases [3]. Lipopolysaccharide (LPS), one of the most well-known pathogen-associated molecules, is produced by Gram-negative bacteria [4]. It activates downstream signaling pathways such as mitogen-activated protein kinase (MAPK) signaling that generates pro-inflammatory cytokines, chemokines, nitric oxide (NO), and reactive oxygen species (ROS), all of which are profoundly associated with oxidative stress [5].

Retained oxidative stress can cause various inflammatory diseases due to an imbalance between the production and elimination of ROS [6]. Excessive ROS production leads to inflammatory responses that can damage organelle structure and biomolecules [7]. Therefore, regulating ROS production and enhancing cellular antioxidant systems are crucial for maintaining the cellular balance [8]. Nuclear factor erythroid 2-related factor 2 (Nrf2) [8] is a transcription factor that regulates the expression of antioxidants and protects against the oxidative stress caused by injury and inflammation [9]. Along with heme oxygenase (HO-1), it is involved in anti-inflammatory, antioxidant, apoptotic, and other processes [10]. To study the inflammation and oxidative stress responses, macrophages are commonly used with their phagocytic function in innate immunity [11].

*Haematococcus pluvialis* is a green microalga known for its rich bioactive content, including carotenoids, proteins, and fatty acids [12]. Among these, astaxanthin is an influential antioxidant that makes *H. pluvialis* a high-potential antioxidant microalga [13]. This compound is specifically effective at scavenging free radicals and protecting cells from oxidative damage, both of which are pivotal in inflammatory conditions such as sepsis [14]. Astaxanthin modulates oxidative stress through its unique molecular structure [15]. Additionally, it can remove ROS more effectively than many other antioxidants [16]. However, the poor solubility of astaxanthin in water limits its commercial use [17]. Therefore, various approaches have been reported to increase the solubility and stability of astaxanthin [18]. One such method is the use of hot-melt extrusion (HME) technology to create new chemical entities [19,20] in drug delivery systems (HME-DDSs). In the present study, HME technology was applied to *H. pluvialis* extract to increase the astaxanthin content through nanocapsules. The extracts were manufactured under two thermal conditions (60 °C and 100 °C), termed ASX-60F and ASX-100F, respectively. The pharmacological effects of HME-DDS-derived *H. pluvialis* extracts (ASX-60F and ASX-100F) were compared to those of the non-technology-applied *H. pluvialis* extract (ASX-C) under conditions of excessive inflammation in an LPS-induced sepsis animal model and LPS-stimulated RAW 264.7 macrophages.

## 2. Results

### 2.1. ASX-60F and ASX-100F Treatments Reduced the Mortality and Pro-Inflammatory Biomarker Expression in LPS-Stimulated Mice

**Figure 1:**
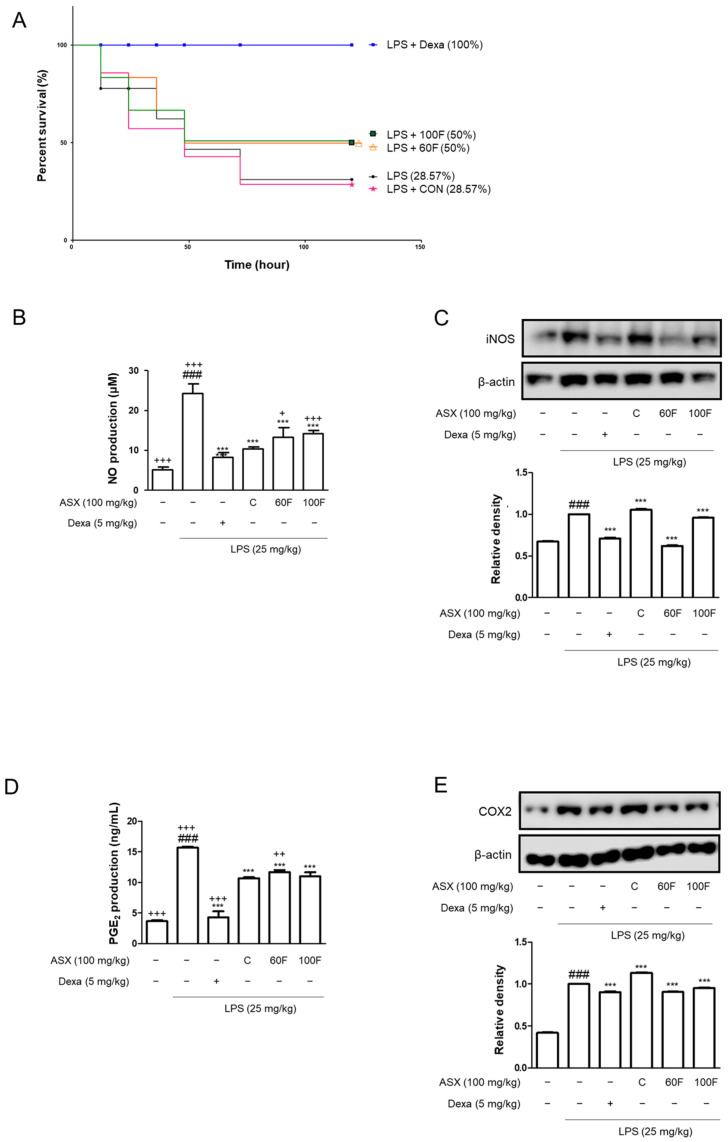
ASX-C, ASX-60F, or ASX-100F reduced mortality and inhibited pro-inflammatory production in mouse models of LPS-induced septic shock. Effects of ASX-C, ASX-60F, or ASX-100F on mortality and inflammatory mediators in LPS-induced sepsis mice model. Mice (*n* = 5~6 per group) were orally administered (per os, p.o.) with ASX-C, ASX-60F, ASX-100F, or Dexa for 1 h followed by 25 mg/kg of LPS i.p. Dexa (5mg/kg, p.o.) stands for dexamethasone (positive control). (**A**) Survival rates of mice monitored for 120 h. (**B**–**E**) Liver tissue obtained from mice 4 h after LPS injection. Production of pro-inflammatory mediators, NO (**B**) and PGE_2_ (**D**); production was measured using EIA enzyme immune assay (EIA). Pro-inflammatory markers, iNOS (**C**) and COX2 (**E**), were determined by using Western blotting. β-actin served as an internal control. Data are shown as mean ± S.D. ### *p* < 0.001 vs. normal; *** *p* < 0.001 vs. LPS; + *p* < 0.05, ++ *p* < 0.01, +++ *p* < 0.001 vs. ASX-C.

We investigated the effects of ASX-C, ASX-60F, and ASX-100F in an in vivo sepsis model. LPS administration (25 mg/kg, intraperitoneal, i.p.) resulted in a 71.43% mortality within 120 h. Oral administration (per os, p.o.) of 100 mg/kg of ASX-C resulted in a mortality that was equal to 28.57% of the survival rate. The administration of ASX-60F and ASX-100F treatments increased the survival rates to 50%. Additionally, 100% of mice treated with dexamethasone (Dexa.) as a positive control survived for 120 h following LPS injection (Figure 1A). The overexpression of pro-inflammatory cytokines causes extensive tissue damage during septic shock. Therefore, we evaluated the effects of ASX-C, ASX-60F, and ASX-100F on the liver, the primary organ affected. ASX-C inhibited the increased protein expression of the pro-inflammatory mediators, iNOS and COX2, as well as the production of NO and PGE2 in response to LPS injection (Figure 1B–E). Septic mice treated with ASX-60F resulted in increased iNOS protein expression compared to that in the ASX-C treatment group (Figure 1C), and ASX-100F treatment resulted in comparable PGE_2_ production (Figure 1D) and COX2 protein expression (Figure 1E).

### 2.2. ASX-60F and ASX-100F Downregulate Inflammatory Responses via ERK/p-38 MAPK Signaling in LPS-Stimulated Mice

**Figure 2:**
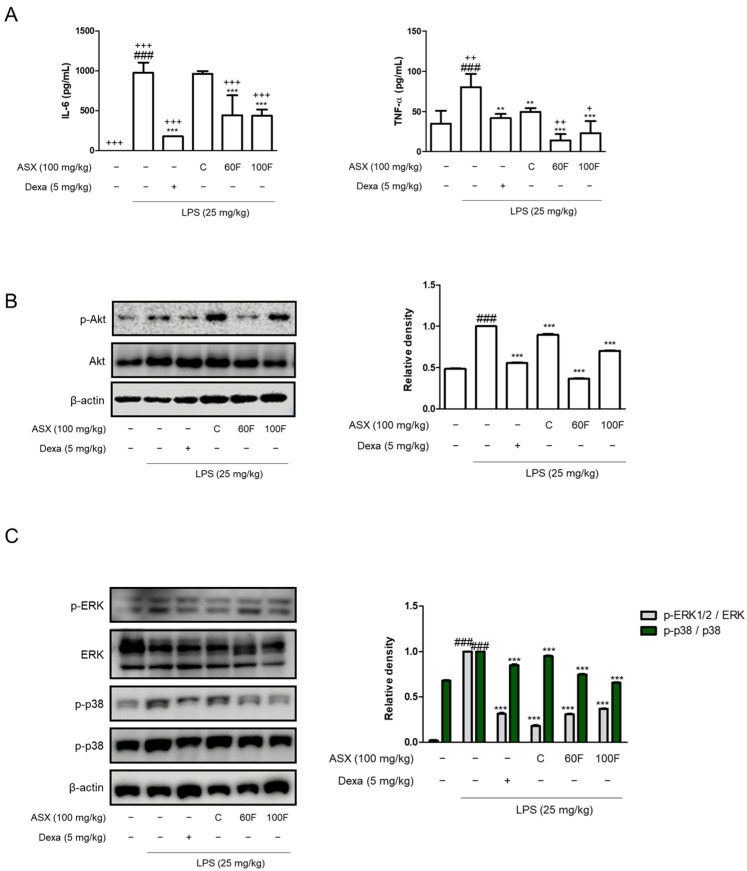
ASX-C, ASX-60F, or ASX-100F reduced pro-inflammatory cytokines and phosphorylation of pro-inflammatory signaling protein expression in mouse models of LPS-induced septic shock. Effects of ASX-C, ASX-60F, or ASX-100F on pro-inflammatory biomarkers in LPS-induced sepsis mice model. Mice (*n* = 5~6 per group) were orally administered with ASX-C, ASX-60F, ASX-100F, or Dexa. for 1 h followed by 25 mg/kg of LPS i.p. (**A**) Pro-inflammatory cytokines were evaluated using enzyme immune assay EIA. (**B**,**C**) Proteins from liver tissue of septic shock mice were determined by using Western blotting assay with β-actin or total form of ERK and p38 as internal controls. Data are shown as mean ± S.D. ### *p* < 0.001 vs. normal; ** *p* < 0.01, *** *p* < 0.001 vs. LPS; + *p* < 0.05, ++ *p* < 0.01, +++ *p* < 0.001 vs. ASX-C.

The interleukin (IL)-6 and tumor necrosis factor-alpha (TNF-α) levels were determined using cytokine assays with enzyme immune reactivity. Both cytokines were significantly decreased by being administered with ASX-60F and ASX-100F compared to those in the LPS-injected septic shock model and ASX-C-treated mice (Figure 2A). The pro-inflammatory markers were increased in the LPS-injected sepsis model via various signaling pathways. One such mechanism involves the phosphorylation of Akt. Increased protein expression was suppressed by the oral administration of ASX-60F and ASX-100F compared to that of ASX-C (Figure 2B). Additionally, we evaluated the effects of ASX-C, ASX-60F, and ASX-100F on the expression of a representative inflammatory signaling pathway such as the MAPK pathway. Macrophages stimulated with LPS exhibited significant phosphorylation of MAPK. ASX-60F suppressed the p38 phosphorylation in septic mice compared to that in LPS-injected or ASX-C-administered mice. ASX-100F also inhibited ERK and p38 phosphorylation compared to LPS-stimulated and positive control groups (Figure 2C). The expressions in the group administered with Dexa showed its role as a positive control (Figure 2).

### 2.3. ASX-60F and ASX-100F Inhibited ERK/p-38 MAPK Signaling in LPS-Induced Inflammatory Responses in Macrophages

**Figure 3:**
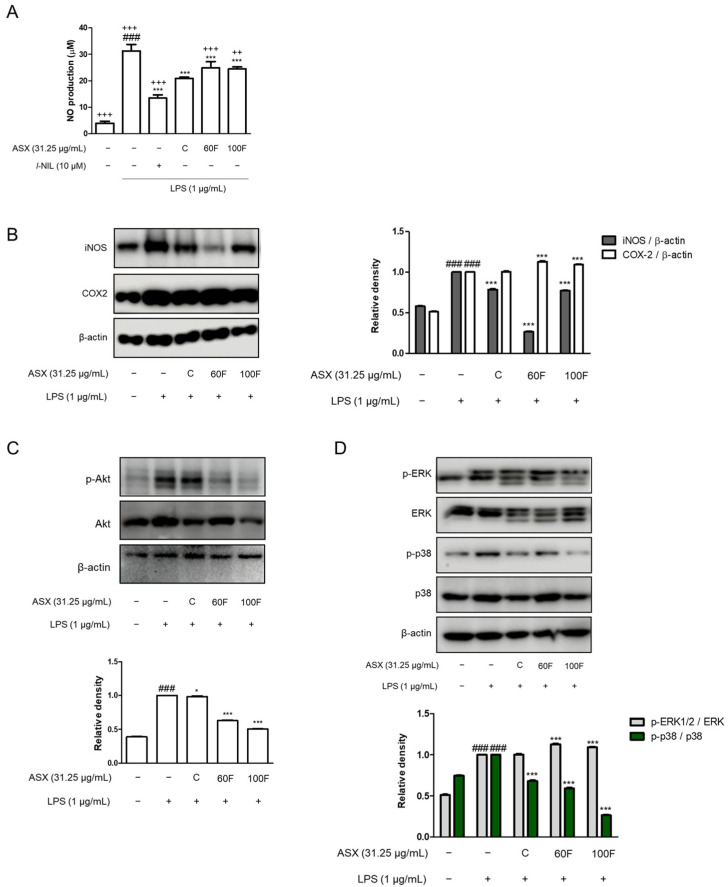
ASX-C, ASX-60F, or ASX-100F suppressed the expression of inducible enzyme and mediators via Akt-ERK/p38 MAPK signaling pathways in LPS-stimulated RAW264.7 macrophages. Effects of ASX-C, ASX-60F, or ASX-100F on Akt-ERK/p38 MAPK pathways in LPS-stimulated RAW264.7 macrophages model. (**A**) Levels of NO were determined using Griess reagent. Positive control was incubated with l-NIL (20 μM). Western blots of iNOS and COX2 (**B**); phosphorylation of Akt (**C**), ERK, or p-38 (**D**) was determined as protein expressions with β-actin or total form of Akt, ERK, or p38 as internal controls. Data are shown as mean ± S.D. ### *p* < 0.001 vs. normal; * *p* < 0.05, *** *p* < 0.001 vs. LPS; ++ *p* < 0.01, +++ *p* < 0.001 vs. ASX-C.

**Figure 4:**
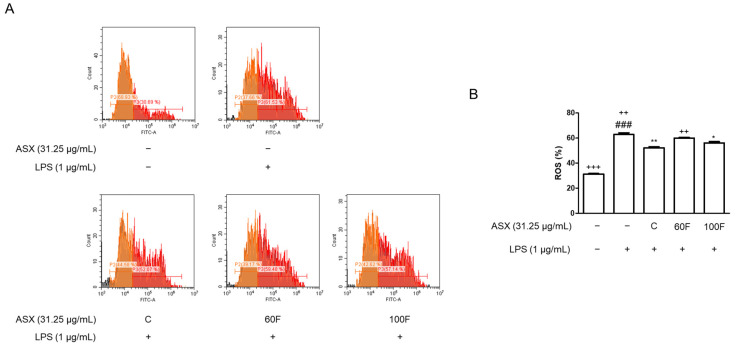
Effects of ASX-C, ASX-60F, or ASX-100F on ROS-generation in LPS-stimulated RAW264.7 macrophages model. (**A**) Cells were pre-treated with or without indicated materials (ASX-C, ASX-60F, or ASX-100F) for 1 h and incubated with LPS for 2 h, and the levels of ROS were measured using FACS. (**B**) Graph indicates relative percentages of ROS-positive cells. Data are shown as mean ± S.D. ### *p* < 0.001 vs. normal; * *p* < 0.05, ** *p* < 0.01 vs. LPS; ++ *p* < 0.01, +++ *p* < 0.001 vs. ASX-C.

**Figure 5:**
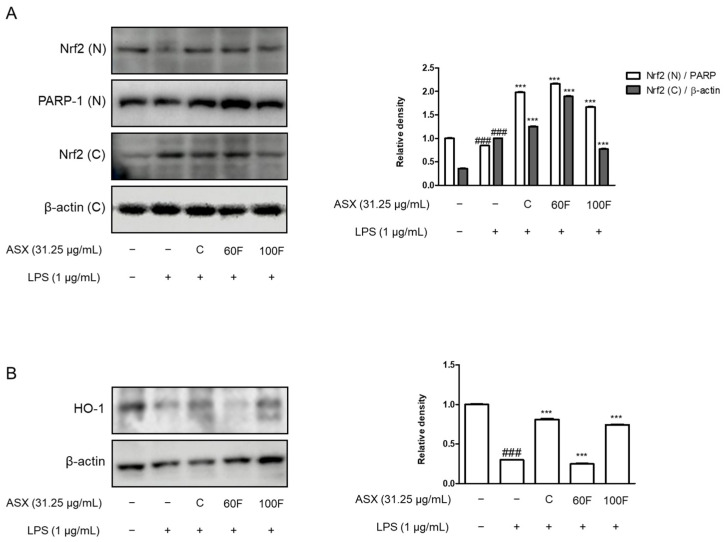
Effects of ASX-C, ASX-60F, or ASX-100F on Nrf2 and HO-1 protein expressions in LPS-stimulated RAW264.7 macrophage model. Cells were pre-treated with or without indicated materials (ASX-C, ASX-60F, or ASX-100F) for 1 h and incubated with LPS for 12 h. (**A**) Nrf2 and (**B**) HO-1 protein expressions were measured by using Western blotting assay. β-actin and PARP-1 served as internal controls for cytosolic fraction (C) and nuclear fraction (N), respectively. Data are shown as mean ± S.D. ### *p* < 0.001 vs. normal; *** *p* < 0.001 vs. LPS.

We performed experiments using LPS-stimulated RAW 264.7 macrophages (Figure 3, Figure 4 and Figure 5). LPS-stimulated macrophages exhibited increased NO production that was downregulated via ASX-C, ASX-60F, and ASX-100F treatment. *l*-NIL served as a positive control in the production of NO (Figure 3A). Consistent with NO production, ASX-C and ASX-100F treatments decreased the protein expression of inducible NO synthase (iNOS) in LPS-stimulated RAW 264.7 macrophages (Figure 3B). However, COX2 protein expression remained unchanged across ASX-C, ASX-60F, ASX-100F, and LPS-stimulated cells (Figure 3B). The phosphorylation of Akt and ERK/p-38 MAPK, which are involved in the representative inflammatory signaling pathways, was observed in the LPS-stimulated cells. The LPS-induced phosphorylation of Akt protein expression was downregulated via ASX-60F or ASX-100F treatments (Figure 3C). The phosphorylation of ERK or p38 was also suppressed via ASX-100F treatment in the LPS-stimulated RAW 264.7 macrophages (Figure 3D).

### 2.4. ASX-60F and ASX-100F Inhibited Excessive ROS Production in LPS-Stimulated RAW 264.7 Macrophages

Previous studies have demonstrated a strong association between oxidative stress and inflammation [2]. To investigate the inhibitory effects of ASX-C, ASX-60F, and ASX-100F on the LPS-induced ROS production in macrophages, we performed an oxidative stress assay using flow cytometry. The LPS-stimulated cells exhibited elevated intracellular ROS levels compared to that of the vehicle-treated normal cells after 2 h. However, the pretreatment with ASX-C, ASX-60F, or ASX-100F inhibited excessive ROS production in the LPS-stimulated RAW 264.7 macrophages (Figure 4).

### 2.5. ASX-60F and ASX-100F Enhanced Nrf2 and HO-1 Protein Expression in LPS-Stimulated RAW 264.7 Macrophages

We determined the protein expression of Nrf2 and HO-1 to observe the effects of ASX-C, ASX-60F, and ASX-100F on the oxidative stress-mediated signaling pathways in the LPS-stimulated RAW 264.7 macrophages. The expression of Nrf2 protein in the nuclear fraction was lower in the LPS-stimulated cells than that in the normal cells. However, ASX-C, ASX-60F, and ASX-100F treatments significantly enhanced this expression (Figure 5A). Additionally, the treatment with ASX-C or ASX-100F upregulated the protein expression of HO-1 in the LPS-stimulated RAW 264.7 macrophages (Figure 5B).

## 3. Discussion

Astaxanthin, a powerful carotenoid derived from *H. pluvialis*, has gained significant attention due to its potent antioxidant and anti-inflammatory characteristics [21]. Due to its poor solubility and low stability, an HME-DDS was applied to *H. pluvialis* extracts (ASX-60F or ASX-100F) to achieve high aqueous solubility and stability, thereby delivering astaxanthin to the target tissues at higher concentrations via nanoencapsulation [22,23]. Prior to targeting the specific tissues, it was necessary to evaluate the systemic effects of these formulations under inflammatory conditions such as sepsis. Although inflammation is necessary to maintain immune homeostasis, excessive inflammation leads to chronic and undesirable phenomena [24].

Sepsis and septic shock are the primary causes of mortality in hospitalized patients, with the dysregulation of the host responses provoking excessive inflammatory damage [25]. In this study, we investigated the effects of ASX-C, ASX-60F, and ASX-100F under LPS-stimulated conditions. To determine the preventative or therapeutic agents for sepsis, we used an in vivo model of LPS-challenged mice [26] and an in vitro model using RAW 264.7 macrophages pretreated with ASX-C, ASX-60F, or ASX-100F [27].

Considering the broad spectrum of LPS dose-dependency according to species, we induced an *E. coli* septic shock model with LPS (25 mg/kg) and observed the survival over 120 h. Mice administered with ASX-C exhibited a 28.57% survival rate after the LPS treatment, similar to that of the LPS-only group. Compared to those results, 50% of the mice treated with ASX-60F and ASX-100F survived after 120 h of LPS injection (Figure 1). The high mortality rate in the ASX-C treatment group appeared to be caused by its low stability or poor solubility in water. Also, the administration of ASX-60F and ASX-100F showed a sustained survival period and higher percentage than other previous studies using astaxanthin [28,29]. The previous studies were conducted using a lower dose of LPS (10 mg/kg or 20 mg/kg) and with pure administration of astaxanthin (40 mg/kg, 100 mg/kg, or 200 mg/kg). The differences in the experimental model show different results suggesting the stability and efficacy of *H. pluvialis* with a higher content of astaxanthin using an HME-DDS.

Following the inflammatory response, we examined the liver and macrophage protein expression of pro-inflammatory mediators. As the largest gland in the human body, the liver plays a critical role in the metabolically and immunologically homeostatic state [30]. And utilizing a sepsis-related liver injury treatment is a presentative strategy to examine a drug delivery system like the HME-DDS [31]. According to the established anti-inflammatory effects, ASX-C treatment exerted inhibitory effects on the production of pro-inflammatory biomarkers including NO, PGE_2_, and TNF-α via the phosphorylation of the Akt-ERK/p38 MAPK pathways. HME-DDS-derived ASX-60F and ASX-100F also suppressed the LPS-induced inflammatory responses in vitro, and in vivo ASX-60F demonstrated a significant suppression of IL-6, TNF-α, Akt protein expression, and p38 MAPK phosphorylation. Meanwhile, ASX-100F exhibited an increased cytokine production and pro-inflammatory mediator expression, such as PGE_2_, compared to ASX-C (Figure 1, Figure 2 and Figure 3). Although the 60F and 100F formulas contain the same ingredients, their manufacturing temperatures appear to induce different results. It is necessary to determine the thermosensitive mechanism underlying these differences [32].

Moreover, astaxanthin has been demonstrated to activate the Nrf2 signaling pathway, thereby enhancing the expression of endogenous antioxidant enzymes such as superoxide dismutase, catalase, and glutathione peroxidase [33]. This activation neutralizes ROS and reduces the overall inflammatory response by suppressing the production of pro-inflammatory cytokines [34]. Interestingly, the ASX-C, ASX-60F, and ASX-100F treatments also upregulated the protein expression of HO-1 in the LPS-stimulated macrophages (Figure 5). As an Nrf2 target gene, HO-1 plays a crucial role in Nrf2-mediated MAPK inhibition [35,36].

According to these results, astaxanthin exerts a dual function, acting as both a direct antioxidant and a modulator of the cellular antioxidant defenses, offering potential benefits for managing oxidative stress-related diseases, such as sepsis [37,38]. Recent studies have explored the potential therapeutic applications of astaxanthin in clinical settings [39]. Its anti-inflammatory and antioxidant properties make it a potent candidate for adjunctive therapy in severe inflammatory diseases like sepsis, where controlling the oxidative stress and inflammation is critical for improving patient outcomes [40,41]. Previous studies have demonstrated that LPS stimulation drives ROS generation, leading to gene alterations in macrophages [42]. Elevated ROS levels typically activate ERKs, JNKs, and p38 MAPK [43]. Considerable data indicate that LPS-stimulated monocytes and macrophages phosphorylate MAPK [44,45,46]. Additionally, the relationship between LPS and Toll-like receptor 4 upregulates gene expression via an ROS/p38 MAPK-dependent pathway [47]. Based on the results of this study, we propose that ASX-C, ASX-60F, and ASX-100F exert ROS-scavenging activity through the regulation of the ERK/p38 MAPK and Nrf2/HO-1 pathways in LPS-stimulated macrophages.

In conclusion, incorporating astaxanthin and HME-DDS formulations, particularly from *H. pluvialis*, into therapeutic strategies may offer a novel approach for mitigating the harmful effects of oxidative stress and inflammation during sepsis. This was shown in Gram-negative bacterial endotoxin, an LPS-induced septic shock mouse model, and an LPS-stimulated RAW264.7 macrophage model. 

## 4. Materials and Methods

### 4.1. Chemicals and Reagents

Dulbecco’s modified Eagle’s medium (DMEM), fetal bovine serum (FBS), penicillin, and streptomycin were obtained from Life Technologies Inc. (Grand Island, NY, USA). LPS (*Escherichia coli* serotype O111:B4 [cat. No. Sigma-Aldrich, L3012]), 3-(4,5-dimethylthiazol-2-yl)-2,5-diphenyltetrazolium bromide, l-N6-(1-Iminoethyl)lysine, N-(2-cyclohexyloxy-4-nitrophenyl)-methanesulfonamide (NS398), and Griess reagent were purchased from Sigma Chemical Co. (St. Louis, MO, USA). Dimethyl sulfoxide was purchased from Junsei Chemical Co., Ltd. (Tokyo, Japan). Primary monoclonal antibodies against COX-2 (sc-1745), PARP-1 (sc-25780), p-ERK (sc-7383), HO-1 (sc-1797), and β-actin (sc-47778) were purchased from Santa Cruz Biotechnology (Dallas, TX, USA). iNOS (cst #2982), ERK (cst #9102), p-p38 (cst#9211), p38 (cst#9212), and Nrf2 (cst#12721) antibodies were obtained from Cell Signaling Technology (Danvers, MA, USA). Horseradish peroxidase-conjugated secondary antibodies and normal goat serum were obtained from Jackson ImmunoResearch Laboratories Inc. (West Grove, PA, USA). Enzyme-linked immunosorbent assay (ELISA) kits specific for IL-6, TNF-α, and PGE_2_ were obtained from R&D Systems (Minneapolis, MN, USA).

### 4.2. Preparation of C, 60F, and 100F (HME-DDS-Applied H. pluvialis) Extracts

*H. pluvialis* DG-1103304 (KCTC14720BP, KCTC, Jeongeup-si, Republic of Korea) was obtained from Jeonbuk National University. The strain was cultured in Optimal Haematococcus Medium, which consists of the following per liter: KNO_3_ (0.41 g), Na_2_HPO_4_ (0.03 g), MgSO_4_∙7H_2_O (0.246 g), CaCl_2_∙2H_2_O (0.11 g), Fe(III)-citrate H_2_O (2.62 mg), SeO2 (50 μg), biotin (25 μg), thiamine (17.5 μg), and vitamin B12 (15 μg). The culture was maintained at 28 °C under fluorescent light with an intensity of 1000 μmol m^−2^s^−1^ for 14 days. Subsequently, the biomass of *H. pluvialis* was harvested via centrifugation (Combi-514R, Hanil, Incheon, Republic of Korea) at 4500 RPM for 30 min and then freeze-dried (FDCF-12015, Operon, Korea). The dried *H. pluvialis* powder contained astaxanthin at a concentration of 4.87 ± 0.54% [48]. The astaxanthin content was analyzed using High-Performance Liquid Chromatography (HPLC, LC-20A, Shimadzu, Japan), with astaxanthin (SML0982; Sigma-Aldrich, St. Louis, MO, USA) for HPLC used as a standard.

**Figure 6:**
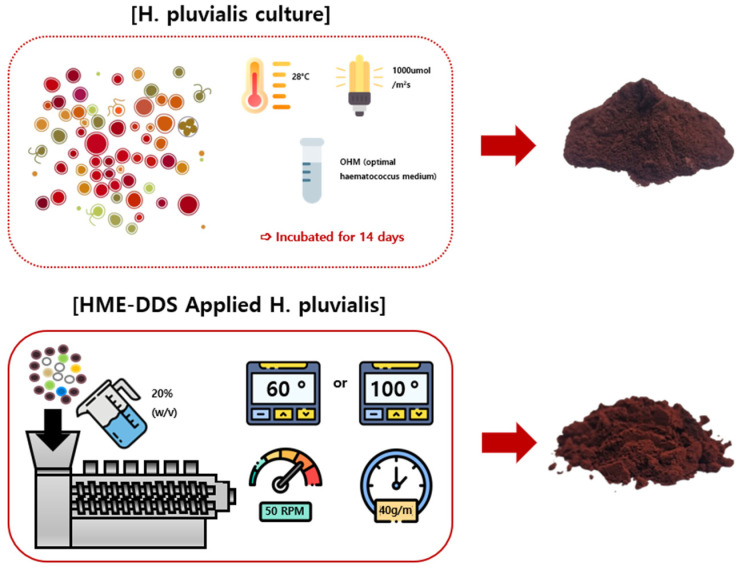
A schematic illustration of *H. pluvialis* production via hot-melt extrusion (HME) process.

The *H. pluvialis* extract (ASX-C; C) was prepared using the HME technique with suitable excipients (Table 1) under two temperature conditions: 60 °C (ASX-60F; 60F) and 100 °C (ASX-100F; 100F). An STS-25HS twin-screw HME (Hankook EM Ltd., Pyoung-Taek, Republic of Korea) was used for this process. To prepare the samples, the extruder was fitted with a circular mold (diameter: 1 mm). The pulverized C was mixed with additives, and 20% (*w*/*v*) distilled water was added to prevent the mixture from overheating during processing. The mixture was then fed into an extruder at a rate of 40 g/min at 50 rpm. The extrusion temperatures were set as follows: 50 → 60 → 60 → 60 → 60 °C (ASX-60F) and 80 → 100 → 100 → 90 → 80 °C (ASX-100F) (Figure 6). After the HME process, the extruded samples were freeze-dried using an FDS-5503 Operon Freeze Dryer (Ilshin Biobase, Dongducheon, Republic of Korea). Samples ASX-C, ASX-60F, and ASX-100F were prepared by drying and grinding the extruded material into powders, which were then dissolved in distilled water for experimental use.

### 4.3. Cell Culture and Sample Treatment

The RAW 264.7 macrophages were purchased from the Korea Cell Line Bank (KCLB, Seoul, Republic of Korea) and cultured in DMEM supplemented with 10% FBS, penicillin (100 U/mL), and 1% streptomycin (100 μg/mL) at 37 °C in a 5% CO_2_ incubator. C, 60F, or 100F extracts were dissolved in distilled water, and cells were treated with 31.25 μg/mL of MF. The cells (1 × 10^5^ cells/mL) were stimulated with 1 μg/mL of LPS for the indicated times, prior to treatment with C, 60F, or 100F extracts for 1 h.

### 4.4. Experimental Animals and Sample Treatment

Six-week-old male C57BL/6 mice were obtained from Daehan Biolink (Eumseong, Republic of Korea). All the animals were housed in accordance with the guidelines for the care and use of laboratory animals. The experimental protocols were approved by the Institutional Animal Care and Use Committee (IACUC) of Kyung Hee University (Approval No. KHSASP-23-442) and complied with the National Institutes of Health guidelines. The mice were housed in a controlled environment (20 ± 5 °C temperature, 40–60% humidity) with a 12 h dark/light cycle and fed standard laboratory chow for one week. They were randomly assigned to one of six groups (*n* = 5–6 per group). The C57BL/6 mice were intraperitoneally (i.p.) injected with phosphate-buffered saline (PBS) or LPS (25 mg/kg dissolved in PBS). Extracts of C, 60F, 100F (100 mg/kg) or Dexa. (5 mg/kg) were administered orally (per os, p.o.) 1 h before LPS injection. Dexa. served as a positive control in the animal model. The survival was monitored for 120 h post-LPS administration. Four hours after LPS injection, peripheral blood and liver samples were collected from each mouse.

### 4.5. NO Production Assay

The NO content was measured indirectly by assaying the culture supernatant for nitrite using Griess reagent that consisted of 1% sulfanilamide in 5% phosphoric acid and 1% α-naphthylamide in H_2_O. The NO production from the macrophages was in the form of NO_2_ in the culture medium. The cells were cultured in DMEM in a 24-well culture plate (1 × 10^5^ cells/mL) and were incubated for 48 h. Cell culture medium (50 μL) was mixed with 50 μL of Griess reagent in a 96-well plate and incubated at room temperature for 15 min, and the absorbance was measurement at 540 nm using an automatic microplate reader (Titertek Multiskan, Lugano, Switzerland). The results are presented as mean ± standard deviation (S.D.) of three independent experiments.

### 4.6. PGE_2_ Assay

The RAW 264.7 macrophages (1 × 10^5^ cells/mL) were treated with C, 60F, or 100F extracts for 1 h before the stimulation with LPS (1 μg/mL). After 24 h, the PGE_2_ levels in the culture medium were measured using a PGE_2_ enzyme immune assay (EIA) kit (R&D Systems, Minneapolis, MN, USA). The experiments were performed in triplicate. Data are presented as mean ± S.D. of the three independent experiments.

### 4.7. Cytokine Assays (TNF-α and IL-6 Production)

The RAW 264.7 macrophages (1 × 10^5^ cells/mL) were pretreated with C, 60F, or 100F extracts for 1 h before the LPS stimulation. Culture media were collected at approximately 24 h post-treatment and stored at −80 °C. The IL-6 and TNF-α levels were measured using specific EIA kits according to the instructions provided by the manufacturer. The values are presented as mean ± S.D. of the three independent experiments.

### 4.8. Nuclear Extraction

The RAW 264.7 macrophages were seeded in 6-well plates (1 × 10^5^ cells/mL) and pretreated with C, 60F, or 100F extracts for 1 h prior to the LPS stimulation. After 12 h, the cells were washed three times with PBS, scraped into 1 mL of cold PBS, and pelleted via centrifugation. The cell pellets were resuspended in hypotonic buffer (10 mM HEPES, pH 7.9, 1.5 mM MgCl_2_, 10 mM KCl, 0.2 mM PMSF, 0.5 mM DTT, and 10 μg/mL aprotinin) and incubated on ice for 15 min. The cells were then lysed by adding 0.1% Nonidet P-40 and vortexed vigorously for 30 min. The nuclei were pelleted via centrifugation at 12,000× *g* for 2 min at 4 °C and resuspended in a high-salt buffer (20 mM HEPES, pH 7.9, 25% glycerol, 400 mM KCl, 1.5 mM MgCl_2_, 0.2 mM EDTA, 0.5 mM DTT, 1 mM NaF, and 1 mM sodium orthovandate). Nuclear extracts from the liver tissue were prepared using NE-PER Nuclear and Cytoplasmic Extraction Reagents (Thermo, Rockford, IL, USA) according to the instructions provided by the manufacturer.

### 4.9. Western Blot Analysis

Protein extracts were isolated from the cell lines (1 × 10^5^ cells/mL) using the protein lysis buffer, Pro-prep^TM^ (Intron Biotechnology Inc., Gyeonggi-do, Republic of Korea). Protein samples were separated on an 8–12% sodium dodecyl sulphate-polyacrylamide gel and transferred to polyvinylidene difluoride membranes. The membranes were blocked with 2.5–5% skimmed milk for 30 min and incubated overnight with specific primary antibodies in Tris-buffered saline (TBS) containing 0.1% Tween20 at 4 °C. The primary antibodies were removed by washing the membrane three times in TBS-T buffer, and the membranes were then incubated for 2 h with horseradish peroxidase-conjugated secondary antibodies (1:2500) at 20–25 °C. After washing three times in TBS-T, the immunoreaction bands were visualized using ECL solution (Absignal, Seoul, Republic of Korea) and detected using X-ray film (Agfa, Belgium). Data are presented as mean ± S.D. of the three independent experiments.

### 4.10. Intracellular ROS Assay

The ROS generation was measured using an oxidative stress kit (Merck Millipore, Darmstadt, Germany) according to the protocol provided by the manufacturer. Briefly, after culture and treatment, the cells were incubated with the oxidative stress working solution, and the proportion of ROS-positive cells was measured using a flow cytometer (Beckman Coulter, Seoul, Republic of Korea).

### 4.11. Statistical Analysis

The results are expressed as the mean ± S.D. from the triplicate experiments. Statistically significant differences were determined using ANOVA and Dunnett’s post hoc test, with *p*-values < 0.05 considered statistically significant.

## Figures and Tables

**Table 1:** Composition and percentage of the contents in the formula.

Composition	Percentage (%)
Astaxanthin	70
HPCD	20
Lecithin	2.5
Ascorbyl Palmitate	5
Vit. C.	1.5
Vit. E.	1
Total	100

## Data Availability

The datasets used and/or analyzed during the current study are available from the corresponding author upon reasonable request.
